# Inhibitory Role of Inducible cAMP Early Repressor (ICER) in Methamphetamine-Induced Locomotor Sensitization

**DOI:** 10.1371/journal.pone.0021637

**Published:** 2011-06-28

**Authors:** Wenhua Han, Yukio Takamatsu, Hideko Yamamoto, Shinya Kasai, Shogo Endo, Tomoaki Shirao, Nobuhiko Kojima, Kazutaka Ikeda

**Affiliations:** 1 Research Project for Addictive Substances, Tokyo Metropolitan Institute of Medical Science, Tokyo, Japan; 2 Tokyo Metropolitan Institute of Gerontology, Tokyo, Japan; 3 Department of Neurobiology and Behavior, Gunma University Graduate School of Medicine, Maebashi, Japan; 4 Laboratory for Neurobiology of Emotion, RIKEN Brain Science Institute, Wako, Japan; Alexander Flemming Biomedical Sciences Research Center, Greece

## Abstract

**Background:**

The inducible cyclic adenosine monophosphate (cAMP) early repressor (ICER) is highly expressed in the central nervous system and functions as a repressor of cAMP response element-binding protein (CREB) transcription. The present study sought to clarify the role of ICER in the effects of methamphetamine (METH).

**Methods and Findings:**

We tested METH-induced locomotor sensitization in wildtype mice, ICER knockout mice, and ICER I-overexpressing mice. Both ICER wildtype mice and knockout mice displayed increased locomotor activity after continuous injections of METH. However, ICER knockout mice displayed a tendency toward higher locomotor activity compared with wildtype mice, although no significant difference was observed between the two genotypes. Moreover, compared with wildtype mice, ICER I-overexpressing mice displayed a significant decrease in METH-induced locomotor sensitization. Furthermore, Western blot analysis and quantitative real-time reverse transcription polymerase chain reaction demonstrated that ICER overexpression abolished the METH-induced increase in CREB expression and repressed cocaine- and amphetamine-regulated transcript (CART) and prodynorphin (Pdyn) expression in mice. The decreased CART and Pdyn mRNA expression levels *in vivo* may underlie the inhibitory role of ICER in METH-induced locomotor sensitization.

**Conclusions:**

Our data suggest that ICER plays an inhibitory role in METH-induced locomotor sensitization.

## Introduction

The inducible cyclic adenosine monophosphate (cAMP) early repressor (ICER) is the collective name for a group of proteins produced from the cAMP response element modulator (CREM)/ICER gene driven by the P2 internal promoter located in an intron of the CREM gene [1]. Lacking the CREM *N*-terminus, ICER only contains two DNA binding domains (DBD I and DBD II) and lacks the activation and kinase-inducible domains. Consequently, ICER functions as an endogenous repressor of transcription of several cAMP response element (CRE)-containing genes [1]–[3]. The P2 promoter of the ICER gene contains two pairs of CRE sequences. The phosphorylated CRE-binding protein (CREB) can induce transcription of the ICER gene from the P2 promoter. The increased ICER competes with CREB in binding with the CRE sequence, blocking transcription from CRE-containing promoters, including ICER's own promoter, and functioning as a potent endogenous CREB antagonist [1], [4].

Four ICER isoforms have been identified: ICER I, ICER Iγ, ICER II, and ICER IIγ. ICER I mRNA contains DBD I and DBD II, but DBD II is absent in the ICER I protein because a stop codon exists at the end of DBD I. The ICER II isoform contains only DBD II. ICER Iγ and ICER IIγ are characterized by a deficiency of exon γ from ICER I and ICER II, respectively [4].

Numerous reports have shown that CREB in the nucleus accumbens (NAc) is associated with responses to drugs of abuse and emotional responses. Chronic drug administration increases levels of CREB immunoreactivity and CRE-binding activity [5]–[6]. Overexpression of CREB by introducing herpes simplex virus-CREB into the NAc decreases behavioral responses to drug administration, whereas blockade of CREB transcription via introducing a dominant-negative CREB mutant or via genetic knockout increases behavioral responses to drug administration [7]–[10]. However, other studies showed that genetic ablation of CREB did not affect the rewarding effects of psychostimulants [11]–[13], indicating that the role of CREB in drug-induced responses is debatable. Recent findings suggest that ICER mRNA expression was threefold higher in the striatum after amphetamine injection [14], suggesting that the endogenous functional CREB antagonist ICER may participate in the mechanisms that underlie the effects of drugs of abuse.

The prodynorphin (Pdyn) peptide is an endogenous ligand of the κ opioid receptor. Cocaine- and amphetamine-regulated transcript (CART) was first sequenced as a peptide with unknown function [15], and previous studies revealed that the CART peptide is co-localized with Pdyn in brain regions associated with drug reward, including the NAc and ventral tegmental area (VTA) [16]–[17]. Both CART and Pdyn play roles as psychostimulant neuromodulators [8], [18]–[20]. CART and Pdyn mRNA are suggested to be CRE-mediated transcripts regulated by CREB *in vitro* and *in vivo* [8], [21]–[23].

Kojima *et al*. [24] generated two types of ICER mutant mice—ICER knockout mice and ICER-overexpressing mice—and suggested a negative role for ICER in regulating long-term fear memory and kindling epileptogenesis. The present study used two types of transgenic mice with opposite genetic alterations of ICER gene expression (i.e., ICER knockout and ICER I-overexpressing mice) and investigated the role of ICER in methamphetamine (METH)-induced locomotor sensitization. Locomotor sensitization is characterized by the progressive enhancement of locomotor activity after repeated psychostimulant exposure [25]–[26]. The augmentation of this behavioral response can be maintained for several months after the cessation of drug treatment [27]. We observed an inhibitory effect of ICER on METH-induced locomotor sensitization. To identify the downstream components of ICER-mediated gene transcription *in vivo* and provide a possible mechanism that contributes to the inhibitory role of ICER in METH-induced locomotor sensitization, we determined METH-induced CREB and phosphorylated CREB (pCREB) levels using Western blot analysis and further determined CART and Pdyn mRNA expression levels in the striatum (caudate putamen [CPu], which mediates locomotor activity) but not in the NAc (which mainly mediates the rewarding effects of drugs of abuse) in ICER I-overexpressing mice and their littermates using real-time reverse transcription polymerase chain reaction (RT-PCR).

## Results

### METH-induced locomotor sensitization in ICER I-overexpressing mice

**Figure 1:**
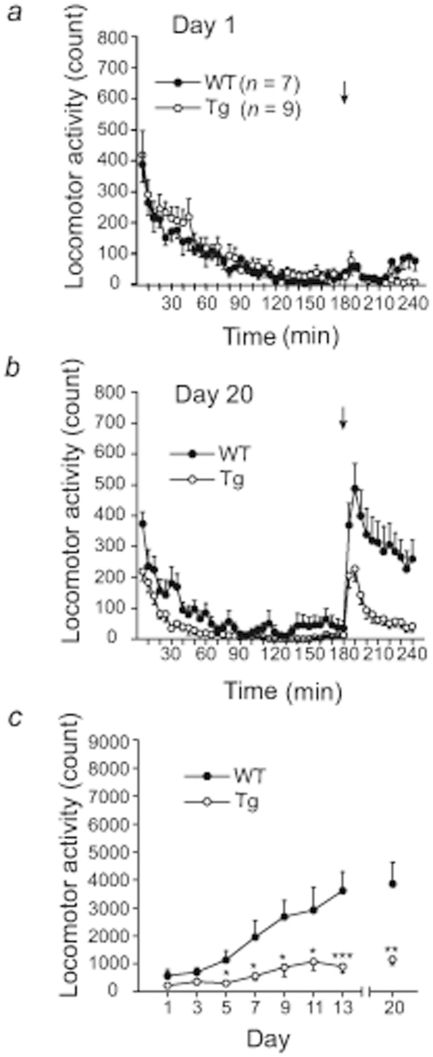
Spontaneous and METH-stimulated locomotor activity in wildtype mice (WT) and ICER I-overexpressing mice (Tg). METH (1 mg/kg) was administered once per day on Days 1, 3, 5, 7, 9, 13, and 20 in WT (*n* = 7) and Tg (*n* = 9) mice. *a*. Time-course of spontaneous locomotor activity before and after METH administration on Day 1. The data are expressed as mean ± SEM beam breaks in 5 min bins. The arrow indicates the start of a METH injection. *b*. Time-course of spontaneous locomotor activity before and after METH administration on Day 20. The data are expressed as mean ± SEM beam breaks in 5 min bins. The arrow indicates the start of a METH injection. *c*. METH-induced locomotor sensitization. The data are expressed as mean ± SEM beam breaks during the 60 min period after METH injection (1 mg/kg) on Days 1, 3, 5, 7, 9, 13, and 20. *p<0.05, **p<0.01, ***p<0.001, significant difference in locomotor activity scores between WT and Tg mice.

Consistent with a previous study [28], on Day 1, the initially elevated levels of locomotor activity in wildtype mice were reduced to near-zero levels after 180 min habituation. ICER I-overexpressing mice displayed a similar pattern of locomotor activity as wildtype mice (Fig. 1*a*). No significant difference in baseline locomotion was observed between genotypes (*n* = 7 for wildtype mice; *n* = 9 for ICER I-overexpressing mice; *F*_1,16_ = 0.49, *p* = 0.49; Fig. 1*a*). On Day 20, ICER I-overexpressing mice displayed decreased levels of spontaneous locomotor activity during the 180 min habituation period compared with wildtype mice (*F*_1,14_ = 9.934, *p* = 0.007; Fig. 1*b*). After a METH injection (1 mg/kg), a significant difference was observed between the two genotypes (*F*_1,14_ = 14.566, *p* = 0.0019; Fig. 1*b*). Repeated administration of METH (1 mg/kg) on Days 1, 3, 5, 7, 9, 11, 13, and 20 significantly increased locomotor activity in both wildtype and ICER I-overexpressing mice (Fig. 1*c*). A two-way, mixed-design analysis of variance (ANOVA; Genotype×Day) revealed a significant effect of Day (*F*_7,98_ = 19.13, *p*<0.0001), indicating the presence of METH-induced locomotor sensitization. METH-induced locomotor sensitization in ICER I-overexpressing mice significantly decreased compared with wildtype mice (*F*_1,14_ = 12.54, *p* = 0.0033; Fig. 1*c*), and a significant Genotype×Day interaction was observed (*F*_7,98_ = 6.52, *p*<0.0001; Fig. 1*c*). From Day 5, locomotor activity in ICER I-overexpressing mice was significantly lower than in wildtype mice (Student's *t*-test).

### METH-induced locomotor sensitization in ICER knockout mice

**Figure 2:**
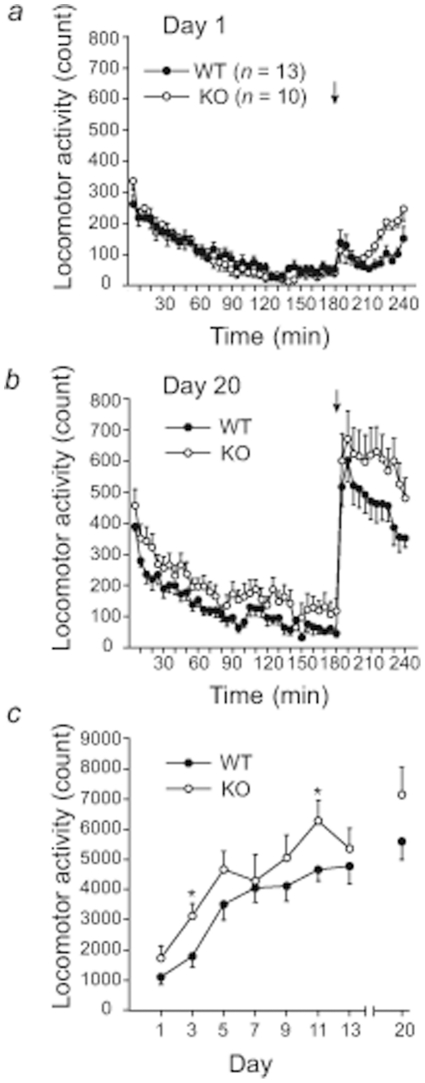
Spontaneous and METH-stimulated locomotor activity in wildtype (WT) and ICER knockout (KO) mice. METH (1 mg/kg) was administered once per day on Days 1, 3, 5, 7, 9, 13, and 20 in WT (*n* = 13) and ICER-KO (*n* = 13) mice. *a*. ime-course of spontaneous locomotor activity before and after METH administration on Day 1. The data are expressed as mean ± SEM beam breaks in 5 min bins. The arrow indicates the start of a METH injection. *b*. Time-course of spontaneous locomotor activity before and after METH administration on Day 20. The data are expressed as mean ± SEM beam breaks in 5 min bins. The arrow indicates the start of a METH injection. *c*. METH-induced locomotor sensitization. The data are expressed as mean ± SEM beam breaks during the 60 min period after METH injection (1 mg/kg). *p<0.05, significant difference in locomotor activity scores between WT and KO mice.

On Day 1, the levels of locomotor activity in wildtype and ICER knockout mice were reduced to near-zero after 180 min habituation. No significant difference in baseline locomotion was observed between genotypes (*n* = 13 for both wildtype and knockout mice; *F*_1,24_ = 0.27, *p* = 0.61; Fig. 2*a*). After repeated procedures on Days 1, 3, 5, 7, 9, 11, and 13 and a 7 day drug-free period, on Day 20, the levels of locomotor activity in the two genotypes were reduced but did not reach near-zero levels after 180 min habituation, which might have been caused by the repeated METH administration. No significant difference was detected between the two genotypes during the habituation period (*F*_1,24_ = 2.731, *p* = 0.12; Fig. 2*b*). After a METH injection (1 mg/kg), locomotor activity in both genotypes increased significantly. No significant difference was observed between the two genotypes (*F*_1,24_ = 2.071, *p* = 0.16; Fig. 2*b*). Repeated administration of METH (1 mg/kg) significantly increased locomotor activity in both wildtype and ICER knockout mice (Fig. 2*c*). A two-way, mixed-design ANOVA (Genotype×Day) revealed a significant effect of Day (*F*_7,168_ = 25.88, *p*<0.0001), indicating the presence of METH-induced locomotor sensitization. ICER knockout mice showed a tendency toward higher locomotor activity compared with their wildtype littermates (*F*_1,24_ = 2.96, *p* = 0.098). ICER knockout mice displayed greater locomotor activity on Day 3 and Day 11 compared with wildtype mice (*p*<0.05; Tukey-Kramer *post hoc* test). No significant Genotype×Day interaction was observed (*F*_7,168_ = 0.62, *p* = 0.74).

### METH-induced CREB expression and phosphorylation in the CPu was abolished in ICER I-overexpressing mice

**Figure 3:**
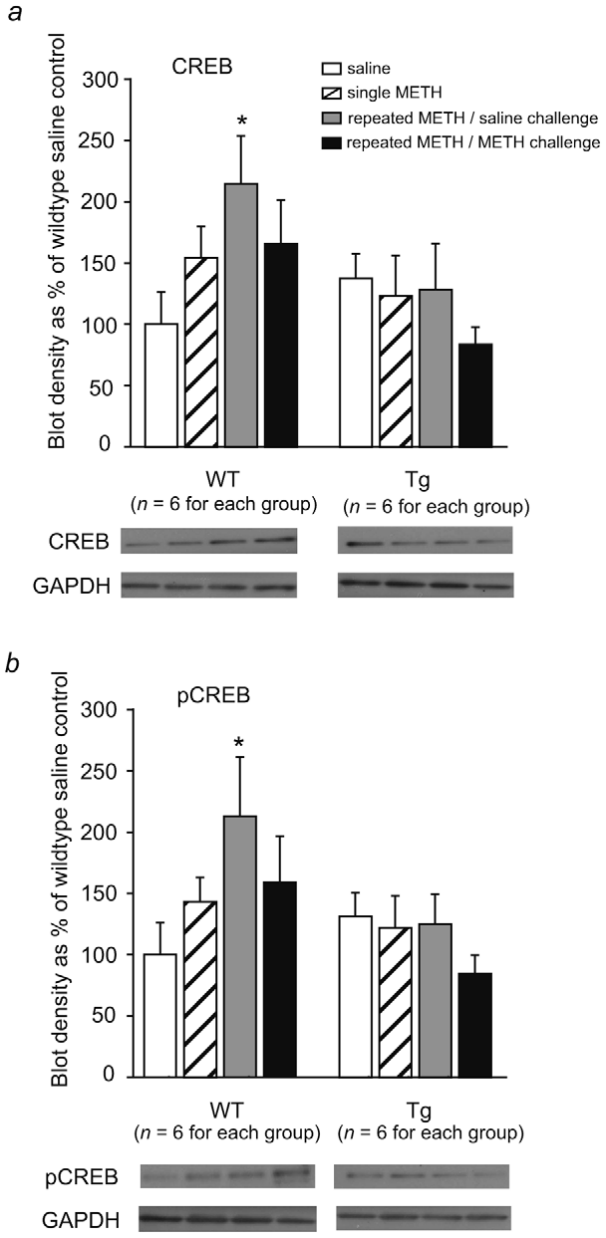
CREB expression and phosphorylation in the CPu after single and repeated METH treatment. The mice were administered METH (1 mg/kg, i.p.) or saline once or received METH (1 mg/kg, i.p.) once every other day from Day 1 to Day 13 and challenged with saline or METH (1 mg/kg, i.p.) on Day 20 after a 7 day drug-free period. The mice were decapitated 1 h after the last METH or saline treatment. The blot density of each group was normalized to that of the wildtype saline group and is expressed as mean ± SEM (*n* = 6). *a*. METH-induced CREB expression in the CPu in wildtype mice (WT) and ICER I-overexpressing mice (Tg). *p<0.05, significant difference in normalized CREB blot density compared with wildtype saline group. *b*. METH-induced CREB phosphorylation in the CPu in wildtype mice (WT) and ICER I-overexpressing mice (Tg). *p<0.05, significant difference in normalized pCREB blot density compared with wildtype saline group.

Two-way ANOVA revealed marginal differences between genotypes in CREB and pCREB protein levels in the CPu after repeated METH treatment (CREB: *F*_1,40_ = 3.76, *p* = 0.06; pCREB: *F*_1,40_ = 3.51, *p* = 0.07). No significant difference in the effect of METH was found (CREB: *F*_3,40_ = 1.28, *p* = 0.29; pCREB: *F*_3,40_ = 1.38, *p* = 0.26), and no Genotype×METH interaction was observed (CREB: *F*_3,40_ = 1.90, *p* = 0.15; pCREB: *F*_3,40_ = 1.79, *p* = 0.16). The Dunnett *post hoc* test revealed that repeated METH/saline challenge significantly increased CREB protein levels in wildtype mice compared with the saline group (*n* = 6 per group, *p*<0.05; Fig. 3*a*). The level of activated CREB protein (pCREB) in the repeated METH/saline challenge group also significantly increased in wildtype mice (*n* = 6 per group, *p*<0.05, Dunnett *post hoc* test; Fig. 3*b*). However, the levels of CREB and pCREB protein were not significantly altered after repeated METH injection in ICER I-overexpressing mice (Fig. 3).

### ICER overexpression significantly reduced CART and Pdyn mRNA expression in the CPu

**Figure 4:**
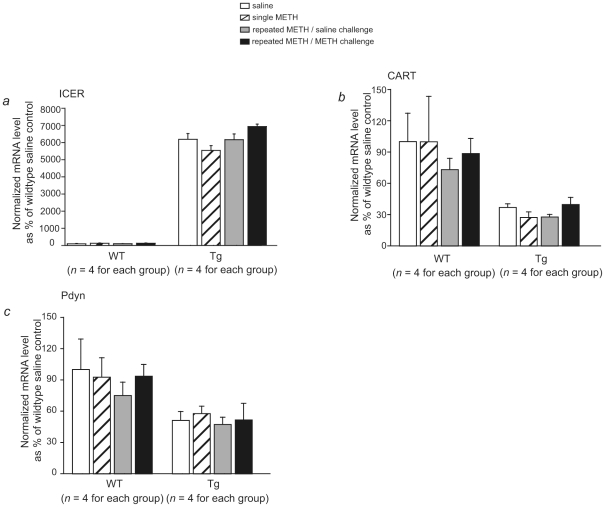
ICER, CART, and Pdyn mRNA levels in the CPu after single and repeated METH treatment. The mice were administered METH (1 mg/kg, i.p.) or saline once or received METH (1 mg/kg, i.p.) once every other day from Day 1 to Day 13 and challenged with saline or METH (1 mg/kg, i.p.) on Day 20 after a 7 day drug-free period. The mice were decapitated 1 h after the last METH or saline treatment. *a*. ICER mRNA expression in the CPu in wildtype (WT) and ICER I-overexpressing (Tg) mice. The data are expressed as mean ± SEM (*n* = 4). *b*. CART mRNA expression in the CPu after single and repeated METH treatment. The data are expressed as mean ± SEM (*n* = 4). *c*. Pdyn mRNA expression in the CPu after single and repeated METH treatment. The data are expressed as mean ± SEM (*n* = 4).

To identify the downstream components of CRE-mediated gene transcription that contribute to reduced METH-induced locomotor sensitization in ICER I-overexpressing mice, real-time RT-PCR was conducted. First, ICER mRNA levels were evaluated using ICER-specific primers. Significant effects were found for Genotype (*F*_1,24_ = 1850.5, *p*<0.001, two-way ANOVA; Fig. 4*a*). However, METH injection did not significantly affect ICER mRNA levels in wildtype mice (*n* = 4 per group, *p*>0.05, Dunnett *post hoc* test). Furthermore, we evaluated CART and Pdyn mRNA levels because they are suggested to be CRE-mediated transcripts and psychostimulant neuromodulators. Although METH did not alter CART or Pdyn mRNA expression in ICER I-overexpressing mice and their littermates (CART: *F*_3,24_ = 0.31, *p* = 0.81; Pdyn: *F*_3,24_ = 0.38, *p* = 0.77; two-way ANOVA), CART and Pdyn mRNA expression levels were significantly reduced in ICER I-overexpressing mice compared with their littermates (CART: *F*_1,24_ = 17.25, *p*<0.01; Pdyn: *F*_1,24_ = 12.21, *p*<0.01; two-way ANOVA; Fig. 4*b*, *c*). No significant Genotype×METH interaction was observed (CART: *F*_3,24_ = 0.21, *p* = 0.89; Pdyn: *F*_3,24_ = 0.17, *p* = 0.92).

## Discussion

The present study investigated the role of ICER in long-lasting METH-induced behavioral alterations by evaluating METH-induced locomotor sensitization in ICER knockout and ICER-overexpressing mice. The major findings of the present study were that ICER I overexpression significantly inhibited METH-induced locomotor sensitization and blocked METH-induced increases in CREB and pCREB protein levels. Additionally, CART and Pdyn mRNA expression levels in the CPu were significantly reduced in ICER-overexpressing mice. ICER knockout mice displayed a tendency toward higher activity after repeated METH administration compared with their wildtype littermates, although no significant difference was detected between ICER knockout mice and their wildtype littermates. Considering the negative regulatory role of CREB in the effects of psychostimulants [18], [29]–[30], the reduction in METH-induced locomotor sensitization in ICER-overexpressing mice may be attributable to reduced CART and Pdyn mRNA expression, rather than attributable to increased CREB and pCREB protein levels.

### Inhibitory role of ICER in METH-induced locomotor sensitization

Although the mechanisms that underlie locomotor sensitization are not fully understood, it is hypothesized to reflect neuronal adaptations in several brain regions, including in dopamine neurons and the CPu [25]. In the present study, ICER I-overexpressing mice exhibited a significant reduction in METH-induced locomotor sensitization compared with wildtype mice (Fig. 1*c*), whereas ICER knockout mice showed a minimal enhancement of METH-induced locomotor sensitization compared with wildtype mice (Fig. 2*c*). Altogether, these results suggest that ICER plays an inhibitory role in METH-induced locomotor sensitization.

CREB overexpression in the NAc reportedly decreased cocaine- and morphine-induced conditioned place preference (CPP), and decreased CREB in the NAc increased cocaine- and morphine-induced CPP [7]–[8], suggesting that increased CREB in the NAc has an inhibitory effect on the induction of CPP. However, recent studies have reported conflicting results, in which genetic ablation of CREB did not affect the rewarding properties of psychostimulants [11]–[13]. Similarly, some studies demonstrated an inhibitory role of CREB in cocaine-induced sensitization [31]–[32], whereas other studies with CREB mutant mice suggested either minor effects [33] or no effects [11] of CREB on cocaine-induced sensitization. In the present study, overexpression of the endogenous CREB repressor ICER inhibited METH-induced locomotor sensitization. Thus, the inhibitory effect of CREB on the psychostimulant-induced response is debatable. A possible explanation for these discrepant results may include the different gene manipulations (i.e., forebrain- or NAc-specific gene manipulation), different drug types (i.e., METH or cocaine/morphine), and different targeted genes (i.e., ICER or CREB).

Enhanced pCREB in the striatum is a molecular marker of neuroadaptations to chronic psychostimulant-induced plasticity [8], [21], [29], [34]–[35]. In the present study, both CREB and pCREB levels increased in wildtype mice after repeated METH injection. The increased CREB and subsequent pCREB induced by repeated METH might homeostatically oppose the effect of METH [29]. However, the repeated METH-induced increases in CREB levels were blocked by ICER I overexpression, suggesting that the negative regulation of the CREB pathway was absent in ICER I-overexpressing mice. Therefore, the CREB pathway may not be involved in the reduced locomotor sensitization observed in ICER I-overexpressing mice. Additionally, ICER expression was 60-fold greater in ICER overexpressing mice than in wildtype mice, which may not occur under physiological conditions. The 60-fold increase in expression may interfere with the CREB signaling pathway and homeostatic regulation of CREB.

### Inhibitory role of ICER in regulating CART and Pdyn mRNA expression

CART and dynorphin are peptidergic neurotransmitters expressed in the CPu and other brain regions and modulate the rewarding effects of drugs of abuse [17], [26], [36]. CART's involvement in the actions of psychostimulants was first noted in a study that demonstrate that acute cocaine and amphetamine upregulated CART mRNA in the rat brain [37]. However, this report has been controversial because this finding has been difficult to replicate [38]–[40]. Other studies found that binge cocaine exposure, rather than acute administration, reliably increases CART expression [38], [41]. Additionally, Pdyn mRNA has been reported to increase or not change in response to binge cocaine administration [42], [43]. In the present study, neither acute nor repeated administration of METH (1 mg/kg) altered CART and Pdyn mRNA expression in wildtype mice. Furthermore, METH administration (1 mg/kg) did not alter ICER mRNA expression in wildtype mice. A possible reason for this might be that the 1 mg/kg dose of METH may not have been sufficient to induce detectable alterations of ICER, CART, and Pdyn mRNA. However, CART and Pdyn mRNA expression levels significantly decreased as ICER mRNA levels significantly increased, suggesting an inhibitory role of ICER in CART and Pdyn expression. Both the CART and Pdyn genes contain a CRE site in their promoter regions [21]–[22], and CART and Pdyn mRNA levels are regulated by CREB *in vitro* [21], [44] and *in vivo* [8], [23]. Therefore, as a CRE-mediated gene transcription repressor, ICER may inhibit the expression of CART and Pdyn *in vivo*. Our studies using ICER I-overexpressing mice support this hypothesis.

### CART and Pdyn as neuromodulators of the behavioral effects of psychostimulants

The CART and Pdyn peptides are neurotransmitters expressed in brain regions associated with drug reward, including the NAc and VTA [16]–[17]. Numerous studies have suggested that CART and Pdyn play a homeostatic role in the NAc to oppose the effects of cocaine. For example, pretreatment with Dyn A (1–17) is effective at decreasing striatal dopamine levels and attenuating cocaine-induced CPP in mice [45]. Overexpression of CREB, with resulting increases in Pdyn gene expression, in the NAc has been shown to decrease the rewarding effects of cocaine [8]. Microinjection of CART peptide 55–102 into the NAc blocked the rewarding effects of cocaine and amphetamine [46]–[48]. CREB overexpression increases CART mRNA levels in the NAc and decreases the rewarding effects of drugs [23]. However, studies in knockout mice have reported conflicting results. CART knockout mice exhibited attenuated locomotor sensitization induced by amphetamine [18], and Pdyn knockout mice showed decreased locomotor activity evoked by cocaine [30]. ICER I-overexpressing mice with decreased CART and Pdyn expression levels displayed attenuated METH-induced locomotor sensitization in the present study. These discrepant results among CART and Pdyn studies may be attributable to differences between systemic and NAc-specific downregulation of CART or Pdyn. Further studies are needed to clarify the effects of CART and Pdyn in brain regions other than the NAc.

### Conclusion

The present study suggests that ICER plays an inhibitory role in METH-induced locomotor sensitization. Our results support the modulatory effects of the ICER pathway in regulating the effects of drugs of abuse and provide an incentive for exploring the therapeutic potential of stimulating the ICER pathway in the treatment of drug abuse.

## Materials and Methods

### Ethics statement

The experimental procedures and housing conditions were approved by the Institutional Animal Care and Use Committee (Animal Experimentation Ethics Committee of Tokyo Metropolitan Institute of Medical Science, Approval ID: 11-029), and all animals were cared for and treated humanely in accordance with our institutional animal experimentation guidelines.

### Animals

Wildtype, ICER knockout, and ICER I-overexpressing mice were produced by conventional gene targeting and transgenic methods [24]. Briefly, the P2 exon encoding the 5′ coding sequence of ICER was deleted to generate ICER-specific knockout mice. To generate ICER I-overexpressing mice, the entire coding sequence of cDNA was subcloned into a pNN265 vector, and the promoter for Ca^2+^/calmodulin-dependent protein kinase II α (CaMKIIα) was used to express ICER I in the forebrain. The expression patterns of other CREB/CREM family members are not altered in either ICER knockout mice or ICER I-overexpressing mice. ICER knockout mice and their littermates were produced by heterozygote-heterozygote mating. ICER I-overexpressing mice and their wildtype littermates were produced by mating ICER I-overexpressing mice (line I-19) and C57BL/6 mice (CLEA Japan Inc., Shizuoka, Japan) because C57BL/6 is the genetic background strain of ICER I-overexpressing mice. Only naive male mice were used for the experiments. The mice were housed five per cage in a temperature- (22±2°C) and humidity-controlled (55±5%) environment on a 12 h/12 h light/dark cycle (lights on 8:00 a.m. to 8:00 p.m.). The mice had *ad libitum* access to a standard laboratory diet and water. All animal experiments were conducted during the light phase of the cycle, between 9:00 a.m. and 5:00 p.m.

### Drugs

Methamphetamine hydrochloride (Dainippon-Sumitomo Pharmaceuticals, Osaka, Japan) was dissolved in saline (0.9% sodium chloride) and administered intraperitoneally (i.p.) in a volume of 10 ml/kg.

### Locomotor activity

Locomotor activity corresponding to distance travelled was evaluated in a test chamber (25 cm diameter, 27 cm height) and measured in 5 min bins using digital counters with passive infrared sensors (Supermex system, Muromachi Kikai, Tokyo, Japan). Wildtype littermates of ICER knockout mice (*n* = 13), ICER knockout mice (*n* = 13), wildtype littermates of ICER I-overexpressing mice (*n* = 7), and ICER I-overexpressing mice (*n* = 9) were used. The mice were first habituated to the apparatus for 180 min and then injected with METH (1 mg/kg, i.p.). Locomotor activity was then measured for 60 min after the injection. The procedure was repeated seven times, once every other day from Day 1 to Day 13. After a 7 day drug-free period, locomotor activity was measured again after an injection of METH (1 mg/kg, i.p.) on Day 20.

### Western blot analysis

The experiment involved four groups of ICER I-overexpressing mice and wildtype mice: Saline, Single METH, Repeated METH/Saline Challenge, and Repeated METH/METH Challenge. Saline and METH (1 mg/kg, i.p.) were administered once to Saline and Single METH mice, respectively, and the mice were decapitated 1 h after the injection. The Repeated METH/Saline Challenge and Repeated METH/METH Challenge groups received METH (1 mg/kg, i.p.) once every other day from Day 1 to Day 13 and were challenged with saline and METH (1 mg/kg, i.p.), respectively, on Day 20 after a 7 day drug-free period. The mice were decapitated 1 h after the last METH or saline treatment. The brains were removed in less than 45 s and cooled rapidly in ice-cold saline for 30 s. The CPu was then dissected. The tissue was quickly frozen on dry ice, stored at −80°C, and homogenized in 100 µl phosphate-buffered saline containing protease inhibitors (Roche Applied Science, Mannheim, Germany) and PhosStop phosphatase inhibitors (Roche Applied Science, Mannheim, Germany). The homogenate was diluted to 4 µg/µl with 2× Laemmli buffer, heated to 95°C for 2 min, and loaded (20 µg of protein) onto 5–20% gradient polyacrylamide gels. The proteins from eight groups were loaded onto the same gel and separated at 50 mA for approximately 1 h and then transferred onto polyvinylidene membranes in a semi-dry blotter. Nonspecific protein binding sites were blocked by incubating in Blocking One Solution (Nakalai Tesque Inc., Kyoto, Japan). The membranes were incubated overnight at 4°C with phosphor (Ser133) CREB (pCREB) antibody (1∶2000; Millipore, Billerica, MA, USA). After incubation in secondary antibody (horseradish peroxidase-conjugated goat antibody to rabbit, 1∶50,000; Zymed Labs, South San Francisco, CA, USA) for 1 h, the membrane was treated with chemiluminescent substrate (Millipore, Billerica, MA, USA) and visualized by exposure to Hyperfilm electrochemiluminescence film (GE Healthcare Bio-Sciences, Tokyo, Japan). pCREB blots were stripped with 10% acetic acid solution for 15 min at room temperature. The membranes were reprobed for CREB antibody (1∶2000; Cell Signaling Technology, Tokyo, Japan). Finally, the blots were stripped and reprobed for glyceraldehyde 3-phosphate dehydrogenase (GAPDH) antibody (1∶5000; Santa Cruz Biotechnology, Santa Cruz, CA, USA). The blots were quantified using ImageJ software (National Institutes of Health, Bethesda, MD, USA), and the sizes were compared with prestained molecular-weight standards. Individual CREB and pCREB values were divided by their respective sample GAPDH values to obtain CREB/GAPDH and pCREB/GAPDH ratio values for each sample. The CREB/GAPDH and pCREB/GAPDH ratio values from the wildtype saline group were averaged, and the mean was used as a control value. Therefore, the CREB/GAPDH and pCREB/GAPDH ratio values of each sample were calculated as a percentage of this control.

### Quantitative real-time reverse transcription polymerase chain reaction

The experiment involved four groups of ICER I-overexpressing mice and wildtype mice: Saline, Single METH, Repeated METH/Saline Challenge, and Repeated METH/METH Challenge. The saline and METH treatments, euthanasia, brain dissection, and storage of brain tissues were the same as described above for Western blot. Total RNA was isolated using Trizol reagent (Invitrogen Life Technology, Tokyo, Japan) and converted into cDNA using a SuperScript VILO cDNA Synthesis Kit (Invitrogen Life Technology, Tokyo, Japan). The real-time RT-PCR reaction was conducted using a LightCycler 480 Instrument (Roche Applied Science, Mannheim, Germany). The ICER, CART, Pdyn, and β-actin primers for real-time RT-PCR were the following: ICER (5′-GCTGAGGCTGATGAAAAACA-3′ and 5′-GCCACACGATTTTCAAGACA-3′), CART (5′-CGAGAAGAAGTACGGCCAAG-3′ and 5′-CACACAGCTTCCCGATCC-3′), Pdyn (5′-TTATGGCGGACTGCCTGT-3′ and 5′- CACTCCAGGGAGCAAATCAG3′), and β-actin (5′-CTAAGGCCAACCGTGAAAAG-3′ and 5′-ACCAGAGGCATACAGGACA-3′). Universal Probes #4, #108, #99, and #64 (Roche Applied Science, Mannheim, Germany) were used for ICER, CART, Pdyn, and β-actin, respectively. Amplification consisted of a preincubation step (95°C for 10 min), 45 cycles of denaturation for 10 s at 95°C, and annealing for 30 s at 60°C. Amplification curves were produced to calculate the crossing point at which the fluorescence of a sample rises above the initial lag phase. Absolute quantification analysis was performed using LightCycler 480 software (Roche Applied Science, Mannheim, Germany). Serial dilutions of an external standard with a predefined, known concentration were used to create a standard curve. The standard dilutions were amplified in separate wells but within the same instrument as the target samples. The crossing points of standards and unknown samples were then used to determine the concentration of the target mRNA. ICER, CART, and Pdyn mRNA levels were normalized according to β-actin mRNA levels. The ICER/β-actin, CART/β-actin, and Pdyn/β-actin values from the wildtype saline group were averaged, and the mean was used as a control value. Therefore, the relative expression levels of CART and Pdyn were calculated as a percentage of this control.

### Statistical analysis

The data are expressed as mean ± SEM. The data for the Western blot, real-time RT-PCR, and locomotor sensitization experiments were analyzed by two-way, mixed-design ANOVA and repeated-measures ANOVA followed by the Dunnett *post hoc* test (for the Western blot analysis and real-time RT-PCR experiments) or Tukey-Kramer *post hoc* test (for the locomotor sensitization experiment). Values of *p*<0.05 were considered statistically significant.
